# Cost-effectiveness of care for type 2 diabetes patients in Nopparat Rajathanee hospital and private clinics

**DOI:** 10.1186/1471-2458-14-S1-P12

**Published:** 2014-01-29

**Authors:** Wanida Peerapattanapokin, Nilawan Upakdee, Pudtan Phanthunane, Supasit Pannarunothai

**Affiliations:** 1 Nopparat Rajathanee Hospital, Bangkok 10230, Thailand; 2 Faculty of Pharmaceutical Sciences, Naresuan University, Phitsanulok 65000, Thailand; 3 Faculty of Business, Economics and Communications, Naresuan University, Phitsanulok 65000, Thailand; 4 Faculty of Medicine, Naresuan University, Phitsanulok 65000, Thailand

## Background

This study aimed to evaluate the cost-effectiveness of care for type 2 diabetes patients from provider perspective between Nopparat Rajathanee hospital and private clinics, the subcontractors of the hospital.

## Materials and methods

This was a retrospective economic evaluation study. Type 2 diabetes patients of the universal coverage scheme who visited and followed up at general practice department of Nopparat Rajatanee hospital and the subcontractor private clinics for at least one year were recruited. The patient data were collected from medical records and from hospital computer systems from 1 January to 31 December 2012. Diabetes patients in five subcontractor private clinics were selected by proportional sampling. Effectiveness of care was based on HbA1c ≤ 7%. The costs were calculated from three parts: overhead cost, medicine and medical supply costs, and laboratory costs.

## Results

One hundred and fifteen diabetes patients were recruited from Nopparat Rajatanee hospital, 43 were male (37.4%), 72 female (62.6%) and the average age was 60.04 ±10.58 years. While 145 patients selected from five private clinics, 31 were male (21.4%), 114 female (78.6%) and the average age was 63.57 ±9.78 years. The overhead cost per one outpatient visit at the hospital and clinics was 480 Baht and 310 Baht, respectively. The clinical parameter by the level HbA1c ≤ 7%, patients at the hospital reached the controlled level lower than patients at the clinics (39.7% and 50.0%, respectively). The cost for a good control of type 2 diabetes patients at Nopparat Rajathanee hospital was higher than at the private clinics (13,215 vs. 8,423 Baht per person per year). Nonetheless, the screenings of risk factors for secondary prevention of complications were higher at the hospital (80.0% in lipid profile and creatinine test, and 63.5% in HbA1c), while lower at private clinics (50.0% in lipid profile and creatinine test, and only 46.9% in HbA1c). Moreover, the screenings of micro-albuminuria, eyes and feet examination were lower than 10% in private clinics.

## Conclusions

The private clinics at the community level seemed to be more efficient or more cost-effective than Nopparat Rajathanee hospital in the care of type 2 diabetes patients under the universal coverage scheme. Further studies should explore the long-term effects of secondary prevention screenings which were found too low in private clinics.

